# Humerus Metastasis From Cholangiocarcinoma: A Case Report

**DOI:** 10.4021/gr523e

**Published:** 2013-03-09

**Authors:** Alessandro Federico, Raffaele Addeo, Domenico Cerbone, Patrizia Iodice, Gaetano Cimmino, Luigi Bucci

**Affiliations:** a Gastroenterology Division, Department of Clinical and Experimental Medicine, Second University of Naples, Naples, Italy; b Oncology Unit, “S. Giovanni di Dio” Hospital, ASL Napoli 2Nord Frattamaggiore, Italy; c Dipartimento Assistenziale di Chirurgia Generale, Oncologica e video assistita Universita Degli Studi Federico II, Naples, Italy; d Surgery Unit, “S. Giovanni di Dio” Hospital, ASL Napoli 2Nord Frattamaggiore, Italy

**Keywords:** Cholangiocarcinoma, Bone metastasis, Humerus, Gemcitabine, Zoledronic acid

## Abstract

Cholangiocarcinoma is a malignant disease of the epithelial cells in the intra- and extrahepatic bile ducts. It is the most frequent biliary malignancy. The lack of effective medical treatment makes a radical surgical resection the only therapeutic option. However, frequently Intrahepatic cholangiocarcinoma metastasize in lymphatic chains, including the hepatoduodenal ligament, and it often invades adjacent organs or metastasizes to other visceral organs such as the lungs, bones, adrenal glands, and brain and the prognosis remains poor. We present a case of elderly patient with a severe and progressive pain due to a pathologic fracture of humerus. The medical investigations revealed the presence of an intrahepatic cholangiocarcinoma.

## Introduction

Cholangiocarcinoma (CCA) is a lethal cancer of the biliary epithelium, originating from the liver (intrahepatic), at the confluence of the right and left hepatic ducts (hilar) or in the extrahepatic bile ducts. It is a rare malignancy associated with poor prognosis. CCA is relatively uncommon with an annual incidence of 1 - 2 cases per 100,000 in the Western countries [1]. However, rates have been rising worldwide over the past decades, partly due to advances in diagnostic techniques. There are several established risk factors for CCA, including parasitic infections, primary sclerosing cholangitis, biliary-duct cysts, hepatolithiasis, and toxins. Other less-established potential risk factors include inflammatory bowel disease, hepatitis C virus, hepatitis B virus, cirrhosis, diabetes, obesity, alcohol drinking, tobacco smoking, and host genetic polymorphisms. Greater than 90% of bile duct cancers are well-differentiated and mucin-producing adenocarcinomas. Surgical resection has been the mainstay of curative treatment for CCA. Unfortunately, many patients present with unresectable tumors. This cancer frequently metastasizes to lungs, adrenal glands, brain, lymphatic system and the axial skeleton [2]. For these patients palliative treatment included bypass surgery, endoscopic or percutaneous stenting, photodynamic therapy, intraluminal brachytherapy, and external radiation and systemic therapy, remain the gold standard of treatment [3]. CCA has been shown to be resistant to common chemotherapy. Numerous drugs have been tested alone and in combination. The response rate has been unacceptably low. Gemcitabine has been suggested as an alternative for patients with unresectable CCA; a survival benefit remains to be proven in a randomized controlled trial [4]. Concerning the value of biological agents like sorafenib, which has an effect on HCC, only a few pilot studies are available and the response rate reported so far is not impressive. Here we describe a patient with advanced CCA carcinoma who presented a unusual bone metastasis of humerus.

## Case Report

**Figure 1:**
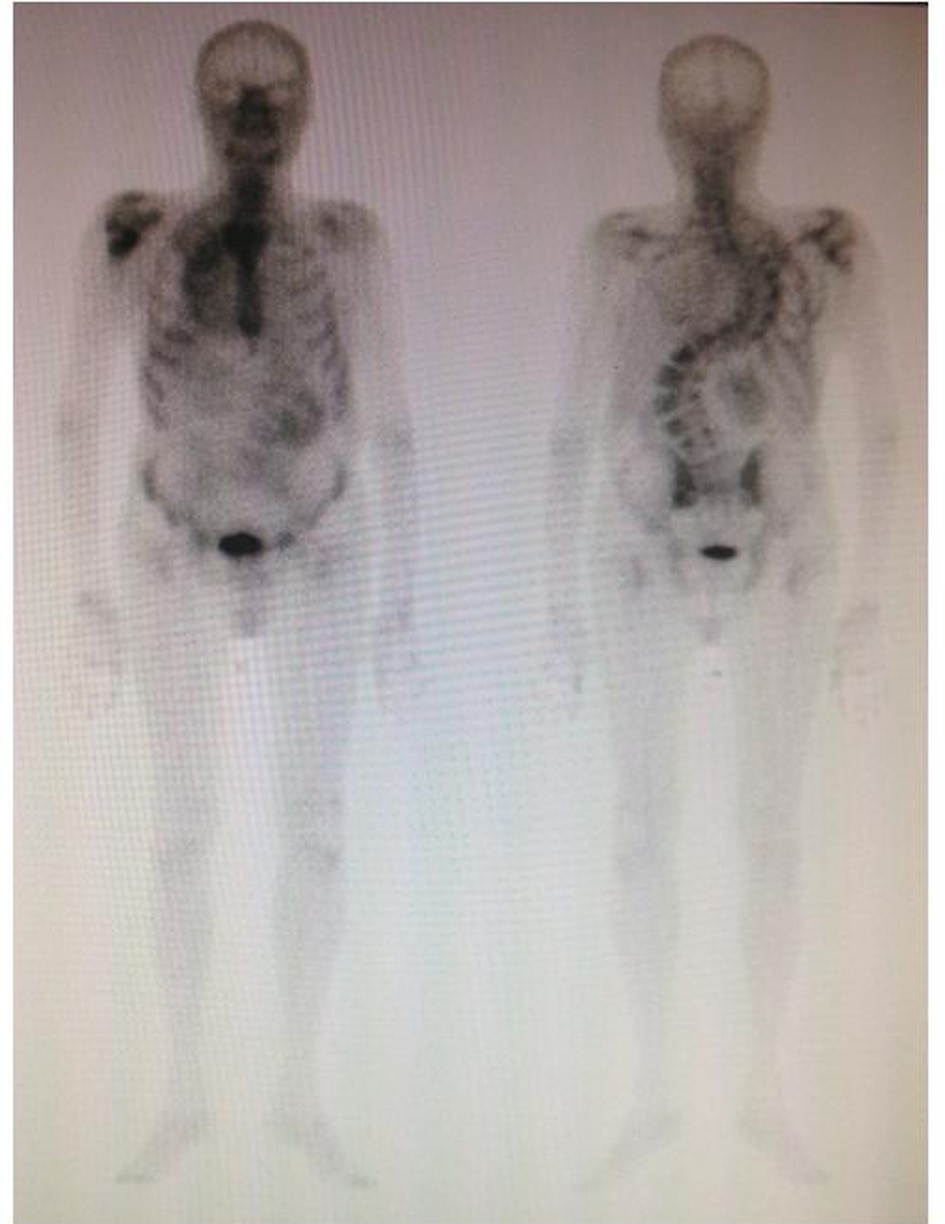
Total body bone scintigraphy showed increased activity in right humerus.

**Figure 2:**
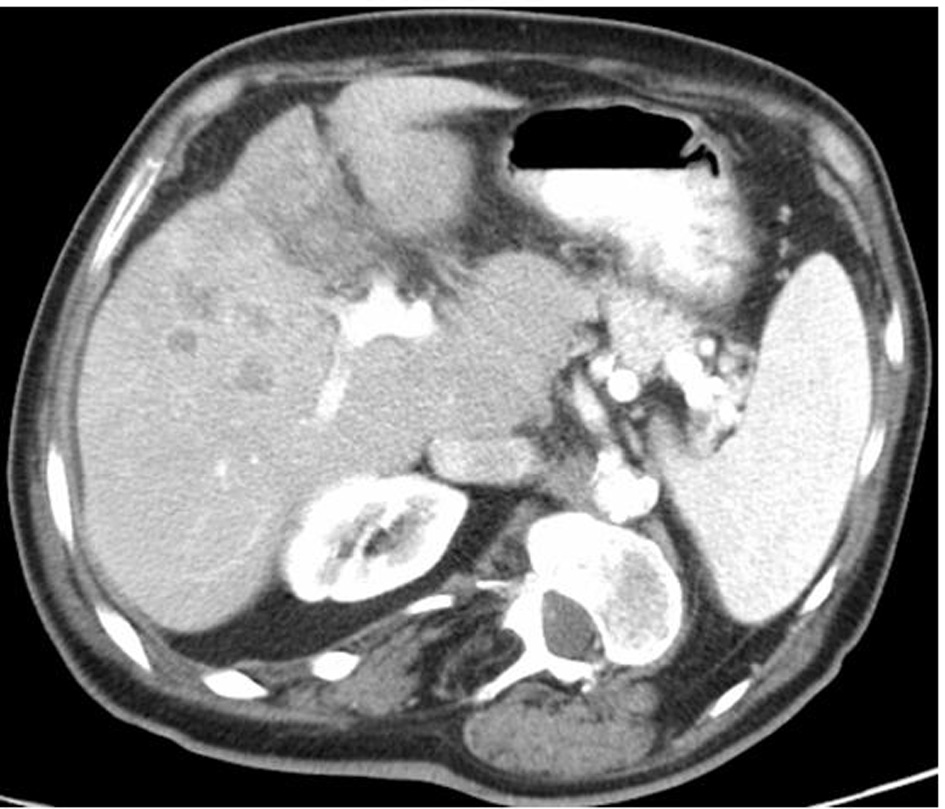
Abdominal computed tomography (CT) findings. Scan revealed several metastatic lesion in the liver parenchyma.

**Figure 3:**
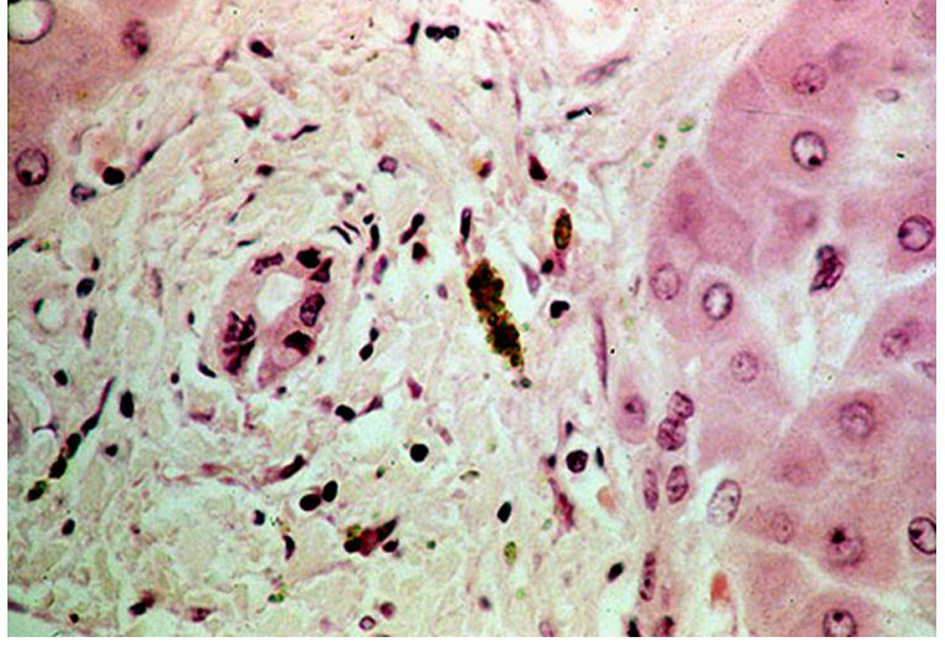
Photomicrograph showing ductal structures with atypical epithelial cells. The pathologic findings revealed an adenocarcinoma that was positive for cytokeratin 7 and 19 but negative for cytokeratin 20 and CEA.

A 71-year-old man with a history of hypertension and diabetes both well controlled, medically and hepatitis C, presented a 2-months history of progressive and severe right homer pain, with asthenia and mild abdominal pain. On physical examination, the patient was not icteric, but had right upper quadrant abdominal tenderness. Hematology and biochemistry laboratory investigations revealed an alteration of liver function: alkaline phosphatase 879 IU/L; Alanine aminotransferase (ALT) 138 IU/L; aspartate aminotransferase (AST) 97 IU/L. The CA19-9 was mildly elevated to 43 (normal level < 37 U/mL).The rest of the other blood tests were within normal limitation. On orthopedic evaluation, X-ray of right shoulder showed an extensive osteolytic lesion of homer compatible with a bone metastasis. Bone scan confirmed the presence of lesion revealing an increased activity in right humerus (Fig. 1). Computed tomography scan (CT) evidenced a solid heteroplastic lesion of 6 centimeters (Fig. 2) and several other nodules with similar morphological characteristics. A biopsy of the liver lesion ultimately revealed the presence of moderately differentiated intrahepatic cholangiocarcinoma. Neoplastic cells were negative for CK-20 and hepatocyte antigen and strongly positive for CK-7 After his diagnosis of cholangiocarcinoma (Fig. 3). The patient subsequently commenced specific therapy with zoledronic acid, and chemotherapy with gemcitabine at 1,000 mg/m^2^ at days 1 and eight, every three weeks. To reduce the strong pain the patient assumed opioids and he effectuated a palliative radiotherapy (30 Gy) on the bone metastasis.

## Discussion

CCA is a lethal cancer of the biliary epithelium and can develop anywhere along the biliary tree. Intrahepatic cholangiocarcinoma is a malignant tumor arising from the epithelial cells of the intrahepatic bile ducts. Intrahepatic cholangiocarcinomas initially present as mass-forming lesions, and obstructive symptoms are rare. The only curative treatment available is surgical management. Unfortunately, many patients present with unresectable tumors, the majority of whom die within a year of diagnosis. In addition to the several well-known risk factors that contribute to CCA, recent studies have investigated the association of ICC with viral hepatitis infections; however their roles as risk factors in ICC development remain unclear. The major metastatic pattern for intrahepatic cholangiocarcinomas is early lymphatic spread. The hepatoduodenal ligament is the most common site for lymph node metastasis irrespective of tumor location. Metastasis is frequent and occurs to many organs, with 50% of patients at autopsy having hematogenous spread to the lung, lymphatic metastasis, and brain, and less frequent to the bone. Metastatic bone disease is common in patients with cancer and is a frequent cause of morbidity in advanced cancer patients. Most patients with bone metastasis suffer from pain resulting from structural damage, periosteal irritation, and nerve entrapment. Pain induced by bone metastases is frequent and difficult to treat because of its intermittent and progressive nature. Recent trial confirmed the benefits of ZA on pain and QoL also in elderly patients with bone metastasis from solid tumors [5]. The development of metastatic lesion of humerus is a rare but possible evolvement of cholangiocarcinoma [6]. However this is the first report that describes a case of cholangiocarcinoma that features with the severe pain due to the pathologic fracture of humerus. This report confirms that the possibility of long bone metastasis should be considered in patients with a history of cholangiocarcinoma.
